# Peritoneal Protein Clearance Is Associated With Cardiovascular Events but Not Mortality in Peritoneal Dialysis Patients

**DOI:** 10.3389/fmed.2022.748934

**Published:** 2022-06-02

**Authors:** Wei Niu, Xiaoxiao Yang, Hao Yan, Zanzhe Yu, Zhenyuan Li, Xinghui Lin, Leyi Gu, Zhaohui Ni, Wei Fang

**Affiliations:** Department of Nephrology, Renji Hospital, School of Medicine, Shanghai Jiao Tong University, Shanghai, China

**Keywords:** peritoneal protein clearance, peritoneal dialysis, cardiovascular events, mortality, endothelial dysfunction (ED)

## Abstract

**Introduction:**

Association of peritoneal protein clearance (Pcl) with outcomes in patients with peritoneal dialysis (PD) is uncertain. Thus, we aimed to investigate its impact on cardiovascular events and all-cause mortality in patients with PD and factors associated with Pcl.

**Methods:**

Prevalent patients with PD from January 2014 to April 2015 in the center of Renji Hospital were enrolled. At the time of enrollment, serum and dialysate samples were collected to detect biochemical parameters and Angiopoietin-2-Tie2 system cytokines. Mass transfer area coefficient of creatinine (MTACcr) and Pcl were calculated. Patients were dichotomized into two groups by the median Pcl level (68.5 ml/day) and were followed up prospectively until the end of the study (1 October 2018).

**Results:**

A total of 318 patients with PD [51.2% men, mean age 56.7 ± 14.3 y, median PD duration 31.5 (12.1–57.2) months] were enrolled. Among them, 25.7% were comorbid with diabetes and 28.6% had a history of cardiovascular disease (CVD). After being followed up for up to 43.9 (24.2–50.3) months, 63 had developed cardiovascular events, and 81 patients were died. Among them, the high Pcl group had occurred 39 cardiovascular events and 51 deaths, and the low Pcl group had 24 cardiovascular events and 30 deaths. Kaplan-Meier analysis showed that both the occurrence of cardiovascular events and all-cause mortality were increased in patients with high Pcl. However, after adjusting for important confounders and serum Angiopoietin-2 (Angpt-2) level, Pcl was still an independent risk factor for cardiovascular events [hazard ratio (HR) = 1.006 (1.000–1.012), *p* = 0.038] but not mortality. On multivariate regression analysis, serum albumin, MTACcr, and body mass index (BMI) were found to be independently associated with Pcl.

**Conclusion:**

High Pcl is an independent risk factor for cardiovascular events but not all-cause mortality. The prediction of cardiovascular events by Pcl was independent of serum Angpt-2.

## Introduction

Peritoneal dialysis (PD) is an established treatment modality for patients with end-stage renal disease (ESRD). It is well known that cardiovascular disease (CVD) is the leading cause of mortality in patients with PD, and about 40–60% of PD mortality is related to cardiovascular events (1). Therefore, preventing the occurrence of CVD remains one of the main treatment goals for patients with PD.

According to the three-pore model (2), peritoneal protein clearance (Pcl) in patients with PD reflects protein leakage across the large-pore pathway of the peritoneal microvasculature, which is equivalent to large pore fluid flux [Jv(L)]. In the past two decades, it has been reported that peritoneal protein loss is associated with the outcomes of patients with PD (3–8). An attractive explanation for this association is that increased Pcl might reflect systemic endothelial dysfunction and could be a biomarker of cardiovascular events and worse survival. However, other studies were failed to find the association between Pcl and outcomes (9–11). Therefore, the potential relationship between Pcl, endothelial dysfunction, cardiovascular events, and mortality remained controversial. Angiopoietin-2 (Angpt-2) is a biomarker of endothelial dysfunction (12–14). The level of circulating Angpt-2 in patients with PD is higher than that in healthy controls (15) and increased circulating Angpt-2 is a strong predictor of cardiovascular events in patients with PD (16). Whether serum Angpt-2 was involved in the association of Pcl and outcomes of patients with PD was unknown.

Therefore, we conducted this study to investigate the impact of Pcl on cardiovascular events and all-cause mortality in a large prospective cohort of patients with PD and take Angpt-2, a marker of endothelial dysfunction, into account.

## Materials and Methods

### Study Design

This was a prospective observational study. Study participants were recruited from January 2014 to April 2015 in the PD center of Renji Hospital, School of Medicine, Shanghai Jiao Tong University and prospectively followed up to the primary end point or the end of the study (1 October 2018). All procedures in this study were following the ethical standards of the responsible committee on human experimentation and the Helsinki Declaration of 1975. The study protocol was approved by the Human Research Ethics Committee of Renji Hospital, School of Medicine, Shanghai Jiao Tong University. All participants gave their written informed consent.

### Study Participants

Stable patients with PD were eligible for this study. Exclusion criteria included patients with acute coronary syndrome, acute heart failure, peritonitis, exit-site infection, or other infectious complications within 4 weeks of enrollment or with underlying active malignancy, chronic liver disease, systemic lupus erythematosus requiring immunosuppression, systemic vasculitis, chronic rheumatic heart disease, and congenital heart disease; the presence of systemic inflammatory disease; patients who refused to give consent; or patients with incomplete data. Based on the inclusion and exclusion criteria, 318 patients with PD were enrolled in our study. All patients were dialyzed using conventional lactate-buffered glucose-based PD solutions (Dianeal®, Baxter, China).

### Demographic and Comorbidity Data Collection

The following data were collected at the time of enrollment: age, gender, height, weight, PD duration, underlying disease of ESRD, and the presence of comorbidity that includes diabetes and CVD. CVD was defined as the presence of ischemic heart disease, history of angina, previous myocardial infarction, coronary artery bypass surgery or stenting, class III–IV congestive heart failure, cerebrovascular event, transient ischemic attack, or peripheral arterial disease with or without amputation. Diabetes was defined either as a comorbid disease or as the etiology of ESRD. Whether taking angiotensin-converting enzyme inhibitors/angiotensin II receptor blockers (ACEI/ARB) or not was also collected.

### Measurement of Pcl, Solute Clearance, and Membrane Function

Peritoneal protein clearance was calculated by the following equation (17): 24-h dialysate protein loss/(serum albumin/0.4783) and expressed as milliliters of plasma per day. Peritoneal total protein losses were measured in 24-h effluent using the Biuret reaction analytical method. Dialysate protein loss was measured by pyrogallol red colorimetry [Total Protein UC FS, DiaSys Diagnostic Systems (Shanghai) CO., LTD].

Solute clearance was determined by measuring total weekly urea clearance (Kt/V) and creatinine clearance (Ccr) using standard methods (18). Weekly Ccr was normalized to 1.73 m^2^ of body surface area. In total, 24 h urine and ultrafiltration were recorded. The contribution of urea clearance by PD was estimated separately. For the evaluation of residual renal function (RRF), we used the estimated glomerular filtration rate, calculated as an average of 24-h urine urea and Ccr (19).

At enrollment, a standard peritoneal equilibration test (PET) was performed on each patient. Mass transfer area coefficient of creatinine (MTACcr) and 4 h D/P creatinine ratio were used to evaluate the peritoneal solute transport rate. MTACcr was calculated using the simplified Garred equation based on the data obtained from the PET test (20): MTACcr (ml/min) = Vd/t ln (Vi·P/(Vd (P–Dt))), where Vd is the drained volume, t is the dwell time (240 min), Vi is the instilled dialysate volume, P is the plasma concentration, and Dt is the dialysate concentration at the end of dwell time, normally determined in the dialysate after drainage. A 4h D/P creatinine ratio was the ratio of dialysate creatinine to plasma creatinine at 4 h in PET.

### Cytokines and Biochemical Parameters

At the time of enrollment, the fasting venous blood sample of each patient was collected for the measurement of Angpt-1, Angpt-2, and soluble Tie-2 (sTie-2). Meanwhile, these cytokines in the overnight drained PD effluent were also determined. All of these cytokines were detected using an enzyme-linked immunosorbent assay (ELISA) kit (R&D Systems Inc, Minneapolis, MN, USA). All samples were run simultaneously and in duplicate to avoid intra- and inter-assay variations. The dialysate cytokines levels were influenced by ultrafiltration volume and by peritoneal solute transport rate, so the intraperitoneal cytokines levels were assessed as appearance rate, which was calculated as dialysate concentration times the drained volume divided by the dwell time and expressed as picogram per minute (pg/min). The following biochemical parameters were measured: high sensitivity C-reactive protein (hs-CRP), serum albumin, and brain natriuretic peptide (BNP). hs-CRP was measured by using the Tina-quant CRP (Latex) ultra-sensitive assay (D & P Modular analyzer, Roche Diagnostics GmbH, Mannheim, Germany). Serum albumin was measured using the bromocresol purple method, while BNP was measured using a fluorescence immunoassay with the Triage BNP Test (Biosite, San Diego, CA, USA).

### Patients' Follow-Up

All patients were followed up prospectively from the enrollment of the study until the primary end point, PD cessation, or to the end of the study (1 October 2018). The primary end point included cardiovascular events and mortality from all causes. For patients who developed multiple cardiovascular events, analysis was limited to the first one. Cardiovascular events included acute myocardial ischemic event, sustained atrial or ventricular arrhythmia, class III–IV congestive heart failure, stroke, peripheral vascular disease, and sudden death, which was defined and diagnosed clinically as unexpected natural mortality within 1 h from symptom onset and without a prior condition that would appear fatal.

### Statistical Analysis

Continuous data were expressed as mean ± SD or median (interquartile range) depending on the distribution of data. Patients were stratified by dichotomization of their Pcl level. Demographic features were compared with independent sample *t*-tests, Mann-Whitney U tests, or chi-squared tests, depending on whether the variable was normally distributed, skewed or categorical. Meanwhile, Spearman correlations of Pcl and other variables were performed. Survival and development of cardiovascular events of patients above and below the median value of Pcl were compared by the Kaplan-Meier method and log-rank test. The univariate Cox proportional hazards model was used to identify predictors of cardiovascular events and survival. The factors of *p* < 0.05 in the univariate Cox analysis of cardiovascular events, all-cause mortality, and clinical variables that may affect end point further entered the Cox regression analysis. We plotted scaled Schoenfeld residuals vs. time for all variables and computed their correlation against time to confirm that all variables considered in the Cox regression analysis met the assumptions of proportional hazards. The multivariate linear stepwise regression model analyzed variables that determined Pcl. A value of *p* < 0.05 was considered to be statistically significant. Statistical analysis was performed using SPSS software, version 24.0 (SPSS, Inc., Chicago, IL, USA).

## Results

### Patients' Characteristics

A total of 318 patients were enrolled, which consisted of 163 (51.2%) men with a mean age of 56.7 ± 14.3 y and a median PD duration of 31.5 (12.1–57.2) months. The causes of ESRD were chronic glomerulonephritis in 96 patients (30.2%), diabetic nephropathy in 43 patients (13.5%), hypertensive nephrosclerosis in 10 patients (3.1%), polycystic kidney disease in 9 patients (2.8%), obstructive nephropathy in 4 patients (1.3%), tubulointerstitial nephritis in 4 patients (1.3%), others were 25 patients (7.9%), and the renal diagnosis was unknown in 127 patients (39.9%). Among these patients, 82 patients (25.7%) were comorbid with diabetes and 91 patients (28.6%) had a history of CVD.

### Factor Associations with Pcl

The median Pcl level was 68.5 (52.1–90.3) ml/day in the whole cohort. Patients were dichotomized into two groups by the median of Pcl, namely, those with Pcl < 68.5 ml/day (low Pcl group) and those with Pcl ≥ 68.5 ml/day (high Pcl group). As shown in Tables 1, 2, participants in the high Pcl group are more likely to be older, have a greater proportion of background CVD, with higher BMI, BNP, pulse pressure (PP) lower serum albumin, higher MTACcr, and D/Pcr. Although the high Pcl group had increased total Ccr (*p* = 0.029) and peritoneal Ccr (*p* = 0.023), the Kt/V was not significantly different between the two groups. Besides, the high Pcl group had higher dialysate Angpt-2AR (*p* = 0.002), dialysate Angpt-2/1 ratio (*p* = 0.006), and dialysate sTie2 AR (*p* < 0.001).

**Table 1:** Demographics, biochemical variables, peritoneal transport, local, and systemic Angpt-Tie2 system cytokines in the subgroups dichotomized by peritoneal protein clearance.

**Variables**	**Total**	**Dichotomization of peritoneal protein clearance**		***p*-value**
	**(*n* = 318)**	**Low (*n* = 159)**	**High ( *n* = 159)**	
Peritoneal protein clearance (ml/day) ^**^	68.5 (52.1–90.3)	52.1 (43.0–60.2)	91.5 (78.4–106.6)	<0.001
**Clinical variables**
Age (yr)^**^	56.7 ± 14.3	54.1 ± 15.5	59.2 ± 12.5	0.001
Male gender [*n* (%)]	163 (51.2%)	74 (46.5%)	89 (55.9%)	0.093
BMI (kg/m2)^**^	22.5 (20.5–25.3)	21.7 (20.1–24.9)	23.5 (21.5–26.2)	<0.001
Peritoneal dialysis duration (mo)	31.5 (12.1–57.2)	35.2 (15.2–56.4)	26.1 (23.5–28.7)	0.318
Diabetes [*n* (%)]	82 (25.7%)	35 (22%)	47 (29.5%)	0.125
Background CVD [*n* (%)]^*^	91 (28.6%)	36 (22.6%)	55 (34.5%)	0.019
ACEI/ARB [*n* (%)]	168 (52.8%)	78 (49.1%)	90 (56.6%)	0.178
CAPD [*n* (%)]	265 (83.3%)	137 (86.2%)	128 (80.5%)	0.114
Pulse pressure (mmHg)^**^	53 (42–62)	49 (38–57)	56 (48–67)	<0.001
**Biochemical variables**
Serum albumin (g/L)^**^	37.4 (34.2–40.4)	39.6 (36.3–41.9)	36.4 (32.7–38.6)	<0.001
hs–CRP (mg/L)	2.1 (0.78–6.31)	1.92 (0.72–5.58)	2.96 (0.80–7.15)	0.226
BNP (pg/ml)^**^	81.0 (37.0–173.5)	65.5 (33.2–107.0)	142.0 (67.8–319.2)	<0.001
**Dialysis variables**
24h Urine (ml/day)	300.0 (0–800.0)	300.0 (0–837.5)	300.0 (0–700.0)	0.625
24h Ultrafiltration (ml/day)	434.2 ± 553.9	430.0 ± 535.5	438.4 ± 573.4	0.893
Residual renal function (ml/min/1.73 m^2^)	0.920 (0–2.908)	0.915 (0–3.078)	0.980 (0–2.890)	0.531
Peritoneal Kt/V urea	1.56 ± 0.37	1.56 ± 0.35	1.56 ± 0.39	0.916
Renal Kt/V urea	0.24 (0–0.64)	0.20 (0–0.66)	0.20 (0–0.60)	0.687
Total Kt/V urea	1.90 (1.68–2.14)	1.92 (1.69–2.14)	1.86 (1.66–2.14)	0.468
Peritoneal Ccr (L/week/1.73 m2)^*^	42.07 (36.62–49.69)	41.07 (36.07–47.36)	44.29 (37.60–51.91)	0.023
Renal Ccr (L/week/1.73 m2)	9.60 (0–32.12)	9.56 (0–32.93)	11.47 (0–31.49)	0.682
Total Ccr (L/week/1.73 m2)^*^	57.07 (49.81–70.80)	54.19 (47.59–69.33)	60.26 (50.78–73.03)	0.029
MTACcr^**^	7.60 (6.06–9.81)	6.59 (5.45–8.21)	8.38 (6.37–11.46)	<0.001
4h D/P creatinine ratio^**^	0.58 (0.52–0.64)	0.64 (0.57–0.74)	0.61 (0.54–0.69)	<0.001
**Cytokine variables**
Serum Angpt-1 (ng/ml)	42.01 (28.30–64.35)	47.71 (32.80–69.31)	39.68 (29.37–66.40)	0.115
Serum Angpt-2 (ng/ml)	5.44 (3.41–7.85)	5.25 (3.32–7.46)	5.42 (3.56–8.10)	0.522
Serum Angpt-2/1 ratio	0.133 (0.076–0.205)	0.124 (0.069–0.202)	0.148 (0.084–0.207)	0.148
Serum sTie-2 (ng/ml)	20.02 (15.22–24.91)	20.16 (15.03−24.84)	19.88 (15.26–24.59)	0.798
Dialysate Angpt-1AR (pg/min)	408.8 (212.2–723.7)	364.4 (209.8–731.1)	414.2 (207–671.5)	0.907
Dialysate Angpt-2AR (pg/min)^**^	880.5 (473.0–1440.5)	810.1 (487.2–1212.2)	1229.0 (634.1–1666.0)	0.002
Dialysate Angpt-2/1 ratio^**^	2.21 (1.29–3.77)	1.93 (1.17–3.25)	2.63 (1.39–3.93)	0.006
Dialysate sTie-2AR (ng/min)^**^	0.45 (0.33–0.69)	0.40 (0.31–0.60)	0.56 (0.37–0.78)	<0.001
Plasma/dialysate of Angpt-1	275 (167, 641)	295 (171–751)	247 (160–608)	0.192
Plasma/dialysate of Angpt-2^**^	19 (12, 30)	26 (14–33)	16 (10, 29)	0.002
Plasma/dialysate of sTie-2^**^	118 (76, 190)	144 (90–211)	98 (69, 158)	<0.001

*BMI, body mass index; CVD, cardiovascular disease; ACEI/ARB, angiotensin-converting enzyme inhibitors /angiotensin II receptor blockers; CAPD, continuous ambulatory peritoneal dialysis; hs-CRP, high sensitivity C-reactive protein; BNP, brain natriuretic peptide; Kt/V urea, urea clearance index; MTACcr, mass transfer area coefficient for creatinine; Angpt, angiopoietin; Ccr, creatinine clearance; D/Pcr, the dialysate-to-plasma ratio of creatinine*.

* *p = 0.01–0.05;*

** *p < 0.01*.

**Table 2:** Spearman correlations of Pcl and other demographics, biochemical variables, peritoneal transport, local, and systemic Angpt-Tie2 system cytokines.

	***r***	***P*-value**
Age^**^	0.219^**^	0.000
BMI^**^	0.245^**^	0.000
Peritoneal dialysis duration (mo)	−0.086	0.124
Pulse pressure (mmHg)^**^	0.283^**^	0.000
Serum albumin^**^	−0.484^**^	0.000
hs-CRP	0.063	0.285
BNP (pgml)^**^	0.447^**^	0.000
24h Urine (ml/day)	0.022	0.693
24h Ultrafiltration (ml/day)	0.037	0.511
eGFR (ml/min)	0.033	0.557
MTACcr^**^	0.360^**^	0.000
D/Pcr^**^	0.363^**^	0.000
Peritoneal Kt/V urea	−0.022	0.693
Renal Kt/V urea	0.014	0.797
Total Kt/V urea	−0.046	0.411
Peritoneal Ccr (L/week/1.73 m2)^**^	0.158^**^	0.005
Renal Ccr (L/week/1.73 m2)	0.016	0.774
Total Ccr (L/week/1.73 m2)^**^	0.148^**^	0.008
Serum Angpt-1 (ng/ml)	−0.101	0.073
Serum Angpt-2 (ng/ml)	0.046	0.414
Serum sTie-2 (ng/ml)	0.023	0.679
Dialysate Angpt-1AR (pg/min)	0.037	0.517
Dialysate Angpt-2AR (pg/min)^**^	0.223^**^	0.000
Dialysate sTie-2AR (ng/min)^**^	0.287^**^	0.000

*r, correlation coefficient; BMI, body mass index; CVD, cardiovascular disease; ACEI/ARB, angiotensin-converting enzyme inhibitors/angiotensin II receptor blockers; CAPD, continuous ambulatory peritoneal dialysis; hs-CRP, high sensitivity C-reactive protein; BNP, brain natriuretic peptide; Kt/V urea, urea clearance index; RRF, residual renal function; MTACcr, mass transfer area coefficient for creatinine; Angpt, angiopoietin; Ccr, creatinine clearance; D/Pcr, the dialysate-to-plasma ratio of creatinine*.

** *p < 0.01*.

However, there was no significant difference in serum Angpt-Tie2 system cytokine levels between patients in the two groups. No significant difference was seen in RRF, fluid removal (such as daily urine volume and daily ultrafiltration), PD duration, and proportion of patients who did continuous ambulatory peritoneal dialysis (CAPD) and taking ACEI/ARB (shown in Table 1).

The multivariate stepwise linear regression analysis showed that serum albumin, MTACcr, and BMI were independently associated with Pcl, after adjustment for age, background CVD, BNP, dialysate Angpt-2AR, and PP (shown in Table 3).

**Table 3:** Multivariate associations between peritoneal protein clearance and patient characteristics.

**Variables**	**β**	***p*-value**
Serum albumin (g/l)^**^	−0.346	<0.001
MTACcr^**^	0.184	<0.001
BMI (kg/m2)^**^	0.156	0.003
Pulse pressure (mmHg)	0.100	0.057
Age (yrs)	−0.052	0.385
CVD	0.029	0.595
BNP (pg/ml)	0.064	0.224
Dialysate Angpt2 AR (pg/min)	0.074	0.148

*MTACcr, mass transfer area coefficient for creatinine; BMI, body mass index; CVD, cardiovascular disease; BNP, brain natriuretic peptide; Angpt, angiopoietin*.

** *p < 0.01*.

### Pcl, Cardiovascular Events, and All-Cause Mortality

After follow-up for 43.9 (24.2–50.3) months, 63 patients experienced one or more cardiovascular events. The first cardiovascular events included 29 strokes, 16 acute myocardial ischemic events, 9 sudden death, 5 peripheral arterial disease events, and 4 class III–IV congestive heart failure. During the study period, 81 patients (25.5%) had died. Causes of mortality included 21 strokes, 21 infections (7 peritonitis episodes and 14 other infections), 10 sudden death, 9 acute myocardial ischemic events, 4 gastrointestinal bleeding, 3 malignancies (1 lung carcinoma and 2 stomach carcinoma), 3 heart failure episodes, 2 peripheral arterial disease, and 8 patients with unknown causes.

**Figure 1:**
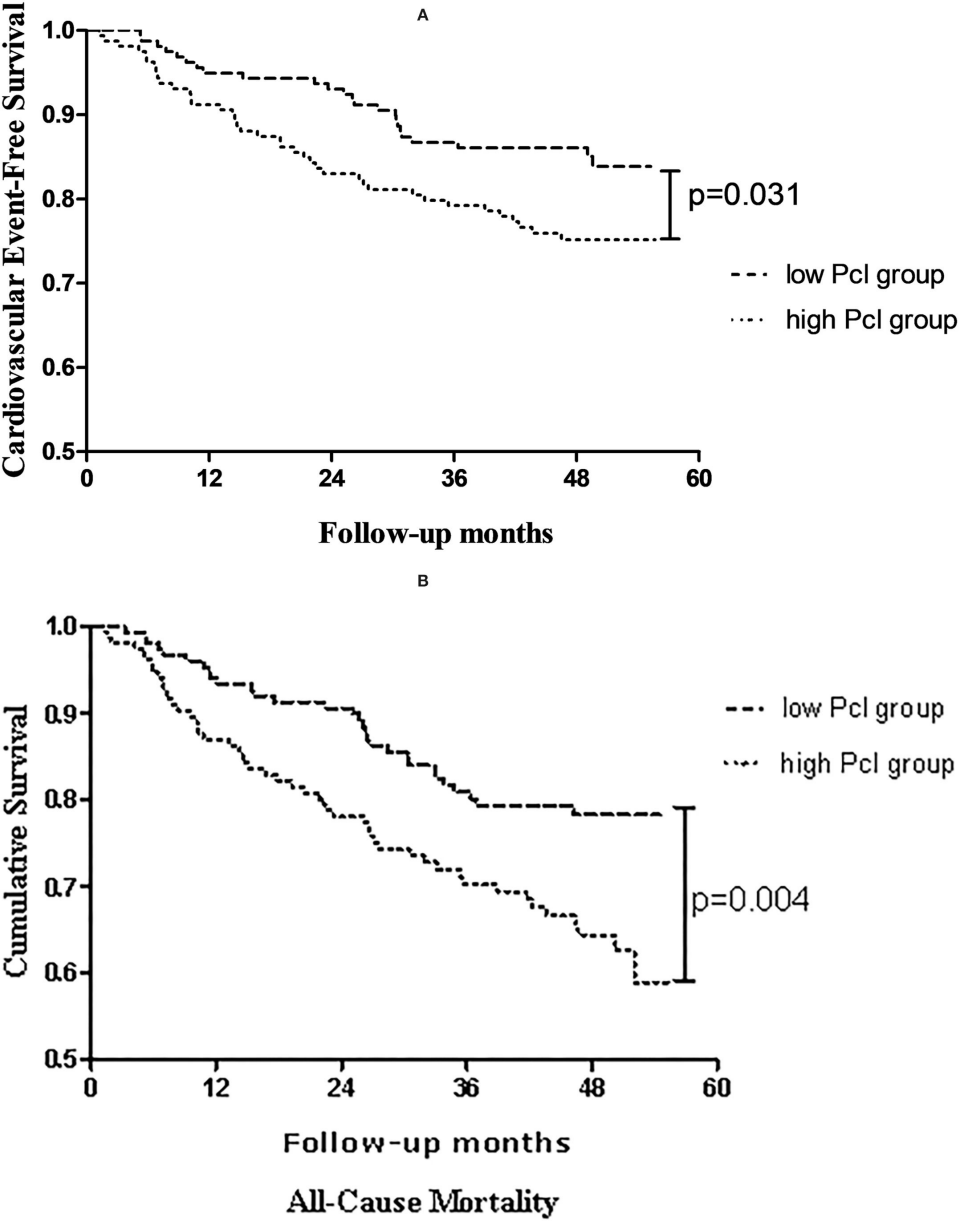
Kaplan-Meier estimates of **(A)** cardiovascular events-free survival, **(B)** overall survival probability of patients stratified by dichotomization of peritoneal protein clearance.

As shown in Figure 1, with comparison to the patients in the low Pcl group, a significant increase in cardiovascular events (log-rank = 4.667, *p* = 0.031; shown in Figure 1A) and all-cause mortality (log-rank = 8.115, *p* = 0.004; shown in Figure 1B) can be observed in patients in the high Pcl group.

After adjusting for age, background CVD, diabetes, BMI, serum albumin, serum Angpt-2, and Pcl remained an independent predictive factor for cardiovascular events [HR = 1.006 (1.000–1.012), *p* = 0.038; shown in Table 4], mainly in patients with PD without diabetes [1.008 (1.002–1.014), *p* = 0.005; shown in Table 5]. In addition, after adjusting for age, background CVD, diabetes, BMI, serum albumin, serum Angpt-2, RRF, and PD duration, the predictive effect of Pcl on all-cause mortality was lost (shown in Table 6).

**Table 4:** Multivariate Cox regression (stepwise backwards) models for cardiovascular events [expressed as hazard ratios, 95% CI's, and p-value].

**Variables**	**HR (95%CI)**	***p*-value**
Background CVD	2.825 (1.633–4.887)	<0.001
Pcl	1.006 (1.000–1.012)	0.038

*Adjusted for age, diabetes, BMI, serum albumin, serum angiopoietin 2; CVD, cardiovascular disease; Pcl, peritoneal protein clearance; BMI, body mass index; Angpt, angiopoietin*.

**Table 5:** Multivariate Cox regression (stepwise backwards) models for cardiovascular events, subgroup analyses stratified by diabetes [expressed as hazard ratios, with 95% CI's, and p-value].

**Variables**	**With diabetes**		**Without diabetes**	
	**HR**	***P*-value**	**HR (95%CI)**	***P*-value**
Background CVD	–	0.377	4.695 (2.493–8.841)	<0.001
Pcl	–	0.858	1.008 (1.002–1.014)	0.005

*Adjusted for age, BMI, serum albumin, serum Angpt2; CVD, cardiovascular disease; Pcl, peritoneal protein clearance; BMI, body mass index; Angpt, angiopoietin*.

**Table 6:** Multivariate Cox regression (enter backwards) models for all-cause mortality [expressed as hazard ratios, 95% CI's, and p-value].

**Variables**	**HR (95%CI)**	***P*-value**
Age (yr)^**^	1.034 (1.013–1.056)	0.002
Background CVD^**^	2.259 (1.569–4.173)	<0.001
Serum albumin (g/l)^*^	0.936 (0.881–0.995)	0.033
Residual renal function^*^	0.822 (0.707–0.956)	0.011
Diabetes	1.129 (0.699–1.822)	0.620
BMI	1.033 (0.968–1.102)	0.329
Pcl	1.001 (0.993–1.009)	0.781
Serum Angpt-2	1.000 (1.000–1.000)	0.485
Peritoneal dialysis duration	0.993 (0.986–1.001)	0.081

*CVD, cardiovascular disease; BMI, body mass index; Pcl, peritoneal protein clearance; Angpt, angiopoietin*.

* *p = 0.01–0.05;*

** *p < 0.01*.

## Discussion/conclusion

In the present study, we found that increased Pcl was an independent risk factor for the occurrence of cardiovascular events but not mortality in patients with PD. The predictive effects of Pcl were independent of serum Angpt-2 level.

In theory, Pcl depends on the effective peritoneal surface area, the density of peritoneal large-pores, and intravascular hydrostatic pressure. According to the three-pore model (2), peritoneal proteins are mainly leaked through large-pore, while MTACcr reflects the small-pore transport of the peritoneum. There are good reasons to believe that the association of Pcl and MTACcr was due to an anatomic coupling, i.e., the increase of effective membrane area is accompanied by an increase in the number of both large-pore and small-pore, consistent with the previous report (6). Similarly, the relationship between Pcl and BMI can be explained by effective peritoneal surface area. The association between PCl and serum albumin is inevitable. First, a large loss of protein will lead to a drop in serum albumin level. Second, the formula for Pcl calculation was used albumin as its denominator. High PP might reflect arterial stiffness and systemic vascular damage (21). We found that high PP also tended to relate to high Pcl despite did not reach statistically significant (*p* = 0.057).

Our study suggested that Pcl is an independent predictor of the occurrence of cardiovascular events in patients with PD. Most of the studies support the conclusion that peritoneal protein leakage may be a manifestation of local or systemic inflammation, reflecting endothelial dysfunction, such as microalbuminuria. Sanchez-Villanueva et al. (22) found an independent association between baseline peritoneal protein losses and peripheral arterial disease. In the present study, the apparent inverse correlation between Pcl and PP seemed to support this notion but the irrelevant between Pcl and serum Angpt-2 denies this view. Our results suggested that the mechanism underlying the association between Pcl and CVD events is not only because Pcl acts as a surrogate for endothelial dysfunction. There might be several possible explanations for this finding: firstly, increased peritoneal protein leakage can cause or aggravate the protein-energy consumption in patients with PD, which is closely related to the occurrence of CVD events (23). In addition, Lee et al. (21) exhibited a positive correlation between Pcl and heart-to-femoral pulse wave velocity implicating arterial stiffness. We also found that high PP, which is a marker of arterial stiffness, tended to be associated with high Pcl. Thus, vascular calcification might play a role. Furthermore, increased protein loss through the peritoneum leads to hypoalbuminemia, which could further cause overhydration. Yoowannakul et al. (24) reported an independent association between Pcl and both extracellular water (ECW)/total body water (TBW) ratio and N-terminal (NT)-pro B-type natriuretic peptide (NT-pro-BNP). Krediet et al. (25) suggested that central venous congestion is the major determinant of Pcl. For patients with long-term PD, overhydration is probably the most important cardiovascular risk factor (26). This study has not yet fully established the relationship between Pcl and systemic vascular dysfunction but confirmed the predictive role of Pcl in cardiovascular events. Clearly, more specific indicators of vascular dysfunction are needed to verify further.

There were several limitations in our study. This was a single-center study and our results might not be generalized to other centers. Pcl was only measured once and the association of longitudinal Pcl change with patients' outcome needs further study. The inclusion of prevalent patients will have skewed the results since patients with short survival or rapid technique failure will not have been included. PD-associated peritonitis was not collected, which might have an impact on Pcl. Inflammatory markers, such as interleukin 6 (IL-6), were not measured in our study. The use of serum albumin instead of total serum protein in the calculation formula of Pcl may introduce more errors. Clearly, multi-center study with a better design is warranted.

In summary, Pcl is independently correlated with serum albumin, MTACcr, and BMI. Increased Pcl is a risk factor for cardiovascular events, but not mortality in patients with PD independent of serum Angpt-2.

## Data Availability Statement

The raw data supporting the conclusions of this article will be made available by the authors, without undue reservation.

## Ethics Statement

The studies involving human participants were reviewed and approved by Ethics Committee of Renji Hospital affiliated to Shanghai Jiao Tong University School of Medicine. The patients/participants provided their written informed consent to participate in this study.

## Author Contributions

WN and XY contributed to the conception of the study, performed the data analyses, and wrote the manuscript. HY, ZY, ZL, and XL helped to collect clinical data. WF, ZN, and LG helped perform the analysis with constructive discussions.

## Funding

This work was supported by the National Nature Science Foundation Grant of China (81370864 and 81670691) and the Shanghai Municipal Education Commission-Gaofeng Clinical Medicine Grant (20152211).

## Conflict of Interest

The authors declare that the research was conducted in the absence of any commercial or financial relationships that could be construed as a potential conflict of interest.

## Publisher's Note

All claims expressed in this article are solely those of the authors and do not necessarily represent those of their affiliated organizations, or those of the publisher, the editors and the reviewers. Any product that may be evaluated in this article, or claim that may be made by its manufacturer, is not guaranteed or endorsed by the publisher.
